# Supplementary material for: Are carbon emissions trading and green financial instruments synergistic? -Comprehensive quantitative research based on content analysis
Source: PLoS One. 2024 Mar 7;19(3):e0298601. doi: 10.1371/journal.pone.0298601 (PMC10919601; doi:10.1371/journal.pone.0298601)
Supplement: S1 Data — (ZIP) [file pone.0298601.s001.zip › Data/地级市Z_F工作报告.zip!/┴╔─■/╕o╦│╩╨/└┤╫╘╣1⁄2╓┌║┼í╛┬φ┐╦╩2╛▌═°í┐_2011.docx]

各位代表：

现在，我代表市政府向大会报告工作，请予审议，并请市政协各位委员提出意见和建议。

一、“十一五”回顾和2010年工作

过去的五年，在省委、省政府和市委的正确领导下，在市人大和市政协的监督支持下，全市人民紧紧抓住东北振兴和沈阳经济区上升为国家战略等历史机遇，励精图治，攻坚克难，全面完成了“十一五”规划确定的各项目标任务。

“十一五”期间，是抚顺抓住机遇、应对挑战、负重前行、加快发展的五年，是城市综合实力不断提升、城乡面貌发生较大变化、人民群众得到实惠增多的五年！预计全市地区生产总值年均增长16%，达到870亿元，是2005年的2.2倍；地方财政一般预算收入年均增长25.3%，达到81.3亿元，是2005年的3.1倍；全社会固定资产投资年均增长37.8%，五年累计2146亿元，是“十五”时期的4.7倍；城市居民人均可支配收入年均增长13.8%，达到15300元，是2005年的1.9倍；农民人均纯收入年均增长13%，达到6883元，是2005年的1.8倍。

这五年，我们明晰了发展思路，与时俱进地提出了“两城两带”和县区跨越发展战略，通过优化城市空间布局，以增量带动产业结构升级，形成了城乡发展的新格局。

这五年，我们全力推进沈抚同城化，举全市之力建设沈抚新城，使其在沈阳经济区37座新城、新市镇中脱颖而出，成为沈阳经济区一体化发展的先导区和抚顺经济增长的新引擎。

这五年，我们加快资源型城市转型振兴步伐，抚矿集团产业转型、抚顺石化改造升级实现历史性突破。五年引进域外资金超千亿元，一大批项目落户抚顺。

这五年，我们着力解决历史遗留问题，国企改革积极推进，改制和进入改制程序的企业数量占应改制企业的98%；在全国率先实施大规模集中连片棚户区改造，惠及城市32万人，10万户家庭圆了住楼梦；集体林权制度主体改革基本完成，配套改革进一步深化，近30万农户受益。

这五年，我们下大气力改善城乡环境，新城区开发与旧城区改造同步推进，劳动公园和儿童公园重新改造，城市集中供热率、自来水和燃气普及率进一步提高，化学需氧量和二氧化硫减排目标提前完成。农村提前两年实现“村村通油路”。

今天的抚顺，发展难题正在逐步破解，发展动能正在迅速聚集，经济已经逐渐走出谷底，开始步入发展的快车道。全市人民关心抚顺、热爱家乡的氛围和情怀与日俱增。我们可以满怀信心地说，抚顺重拾自信、大显身手、再展雄风的时候已经到了！

各位代表：刚刚过去的2010年，是“十一五”的收官之年，也是我们大有收获的一年。一年来，经过全市人民的共同努力，我们全面完成了市十四届人大三次会议确定的各项目标任务，在加快抚顺发展振兴的道路上又迈出了坚实步伐。

（一）沈抚新城建设全面提速

举全市之力建设沈抚新城。调整行政区划，新城面积由118平方公里扩大到229平方公里。完善城市功能，投资14亿元的水系、道路、桥梁、绿化等基础设施建设全面铺开，一批高等院校、医院落户新城。产业区国家先进能源装备产业化基地项目纷纷落地，当年完成投资135亿元；核心区城市综合体项目建设进展顺利；生态区高品质商住和旅游项目渐成规模。沈抚新城已经成为国内外客商关注的焦点、投资的热土。

（二）改革开放取得重大进展

加快推进国企改革，抚顺铝厂、抚顺特钢集团等142户国企进入改制破产程序，以解决职工“一金两险”为重点的138户有遗留问题的国企改革进入收尾阶段。

转变招商方式，主题概念招商、产业集群招商、总部经济招商成效显著，德国汉圣、青岛伊科思、大连万达、上海绿地、山东山推等行业龙头企业相继落户抚顺。全年实际利用外资4.4亿美元，增长42%；引进域外资金410亿元，增长41%。全市超亿元的重点项目完成投资550亿元，增长45%。

积极向上争取，全年争取资金75亿元，同口径增长41.3%；争取建设用地指标840公顷，增长10.5%；获得城乡建设用地增减挂钩周转指标41公顷；争取全国首家矿山地质灾害综合治理试点市工作取得实质性进展。

（三）各种经济发展步伐加快

强化工业优势地位，全年完成工业投资450亿元、增长66%，实现增加值430亿元、增长18%。促进高新技术引进和消化吸收，实现高新技术产业增加值121亿元。以先进能源装备、化工新材料为重点的工业产业集群建设步伐加快。抚顺石化“千万吨炼油、百万吨乙烯”、抚矿集团百万吨造纸和东露天矿恢复生产、抚挖重工大型履带起重机等一批重大项目建设进展顺利。以罕王、方大为代表的民营企业发展迅猛，民营经济总量占全市的比重超过60%。

巩固农业基础地位，培育壮大农业特色产业，设施农业面积达到4400公顷，无公害农产品和绿色食品生产面积达到6.4万公顷。食用菌、中药材和山野菜向适度规模优势产区集中，形成专业乡镇7个、专业村115个。新增畜禽标准化养殖小区230个。新增规模以上农事企业30家，实现销售收入30亿元。

大力实施服务业五年翻番计划，房地产开发势头强劲；天朗国际广场、世城财富广场等一批商贸项目开工建设，新抚中央商务区的框架初步形成；红河峡谷漂流、皇家海洋主题乐园等品牌项目游客火爆，以游、乐、住为主题的丰远·热高乐园项目全面推进，旅游产业集聚区的效应初步显现。

县域和城区竞相发展，完成县域经济三年倍增计划，预计三个县地区生产总值增长24%，地方财政一般预算收入增长54%。实施城区经济三年倍增计划，预计四个城区地区生产总值增长16%，地方财政一般预算收入增长26%。

（四）基础设施和生态环境建设成效显著

抚草高速公路竣工通车，桓永、抚通高速公路续建工程及滨河北路等6项交通重点工程进展顺利。

浑河城市段38.5公里景观带建设正式启动，葛布桥至天湖桥北岸带状公园、人民广场规划建设进展顺利，月牙岛生态公园前期建设全面展开。浑河两岸亮化工程积极推进。

启动国家森林城市创建工作，大规模植树造林，城区植树103万株，新增绿地139公顷，绿化覆盖率达到39.5%。

改善生态环境，建成城镇污水处理厂6座，实施三宝屯、海城、海新河污水处理厂建设及提标改造工程，关闭重污染企业200家，拆除燃煤小锅炉80台，新增城区集中供热面积241万平方米，提前实现主要污染物减排目标。

（五）重点民生工程和社会事业全面推进

多渠道扩大就业，继续保持零就业家庭动态为零，城镇实名制就业10万人，城镇登记失业率控制在4.5%以内。

强力推进扩大社会保险覆盖面、提高缴费率工作，在极其困难的情况下，按时调增、足额发放了29万企业离退休人员的基本养老金。

加大扶贫助困力度，城市低保标准由280元/月提高到330元/月，农村低保标准由1360元/年提高到1661元/年，农村五保对象供养标准由3300元/年提高到3993元/年。实施慈善“助字号”工程，医疗救助患病儿童、老人和精神病患者417人，资助贫困学生2850人，救助贫困村民1239户。新建和改造农村敬老院9所。整村推进扶贫24个村，减少低收入人口4万人。

下大力气解决社会热点、难点问题，拆迁改造棚户区25万平方米、危旧楼22万平方米。对60-69周岁的老年人乘坐公交车、到公立医院看病实施优惠政策，全市17万人受益。投资3400万元，建成饮水工程154处，惠及6万农民。

面对两次特大洪灾，我们迅速组织全市人民抗洪救灾和灾后重建，社会各界伸出援助之手，在最短时间内转移安置群众6万人，在最短时间内全面完成水毁房屋重建和修复，在最短时间内基本修复水毁交通、电力、通信、水利等基础设施，确保了受灾群众有饭吃、有房住、有病能医和学校正常开学，实现了大水大汛无大灾。灾后重建工作受到省政府表扬。

各项社会事业协调发展，市中医院、第四医院、第五医院的改建扩建工程启动实施；新型农村合作医疗参合率达到99%以上，参保补助标准由100元提高到150元。统筹发展各级各类教育，义务教育进一步均衡发展，新建续建中小学13所；抚顺剧院开工建设，市体育馆和煤精琥珀博物馆投入使用。统筹推进人口和计划生育工作，顺利完成第六次全国人口普查入户登记工作。

社会治安进一步好转，群众安全感明显增强，信访总量下降10.8%，安全生产形势总体稳定。

加强农副产品价格监测，建好“菜园子”，抓好“菜篮子”，市场供应充足，物价基本稳定。

工商、质监、药监、国家安全、海关、出入境检验检疫、烟草、金融、保险、邮政、通信、电力、气象、民族、宗教、司法、外事、侨务、监察、审计、统计、人防、档案、供销、防震、老龄、妇女儿童、残疾人等各项工作都取得了新的进展。

（六）政府自身建设取得实效

自觉接受市人大及其常委会的法律监督、工作监督和政协的民主监督，办理人大代表建议391件、政协提案387件，办复率100%，满意率99.7%，解决率56.6%。开通政风行风热线和抚顺民心网,积极接受社会监督。

在全市政府系统开展“讲大局、讲责任、讲效率、鼓干劲，建设人民满意政府”活动，广大干部的工作激情、工作干劲和工作效率进一步提升，政府的执政能力进一步增强。

各位代表：回首刚刚过去的“十一五”，我们深切感受到，我们取得的巨大成就，是省委、省政府和市委正确领导的结果，是市人大、市政协监督支持的结果，是全市人民共同努力的结果。在此，我代表市政府，向人大代表、政协委员，向各民主党派、工商联、无党派人士和人民团体，向离退休老同志，向驻抚人民解放军、武警部队官兵、公安干警和中省直单位，向所有关心、支持抚顺发展的国内外朋友，向全市各族人民，表示崇高的敬意和衷心的感谢!

在肯定成绩的同时，我们也清醒地看到，当前抚顺还存在一些困难和问题。一是产业完整度和丰厚度不够，工业“龙头大、身子小、尾巴短”的问题仍然突出，服务业、新兴产业和民营经济发展还不够充分，项目落地开工率还不高，有重大牵动力的新的经济增长点还不够多。二是国企改革压力依然很大，大集体改革任务十分艰巨，很多遗留问题亟待解决。三是政府债务沉重，集中还贷进入高峰期，财政收支矛盾较为突出。四是城乡居民收入还不够高、增长还不够快，养老保险资金缺口巨大，城镇居民医疗保险覆盖面还不够宽。五是经济发展环境问题依然不少，政府自身建设还有待进一步加强。对此，我们将在今后的工作中着力加以解决。

二、“十二五”主要发展目标和今年重点工作

各位代表：“十二五”时期，是抚顺重振雄风的关键时期，也是大有作为的重要时期。

“十二五”时期全市经济社会发展的指导方针是：以邓小平理论和“三个代表”重要思想为指导，深入贯彻科学发展观，紧紧抓住国家进一步实施东北振兴战略和沈阳经济区上升为国家战略的重大机遇，以科学发展、创新发展、和谐发展为主题，以加快经济发展方式转变和社会管理模式转型为主线，坚持增量带动结构优化、创新促进产业升级、发展保障民生改善，大力实施“两城两带”和县区跨越发展战略，深化改革开放，强化项目建设，改善软硬环境，全面发展各项社会事业，显著提高人民生活水平，基本完成资源型城市向资源深加工型城市转变，初步建成生态型工业强市，基本实现抚顺老工业基地全面振兴。

全市经济社会发展的主要预期目标是：地区生产总值年均增长18%，地方财政一般预算收入年均增长27%，全社会固定资产投资年均增长30%，社会消费品零售总额年均增长18%，实际利用外资年均增长15%，引进域外资金年均增长30%，城市居民人均可支配收入年均增长15%，农民人均纯收入年均增长14%。

全市经济社会发展的重点任务是：加快推进以转方式调结构为重点的经济转型，增强综合经济实力和竞争力；加快推进以保障和改善民生为重点的社会转型，提高人民生活质量和水平；加快推进以消除体制障碍为重点的体制转型，激发经济社会发展的动力和活力。力求速度与质量、效益相统一，科技创新与技术创新、管理创新相统一，经济效益与社会效益、生态效益相统一，城乡之间、区域之间发展相统一，富民与强市相统一。

未来五年，将是抚顺经济开始发力、加快发展的五年。“两城两带”和县区跨越发展战略将凸显成效，全市经济将形成以沈抚新城、石化新城、县域和城区为四大支撑，“千万吨炼油、百万吨乙烯”等一批重大项目竣工投产将形成新的经济增长点。只要我们坚持既定发展思路不动摇，做大经济总量，提高发展质量，抚顺经济就一定能够实现高于“十一五”时期平均发展水平、高于全省平均发展水平的“两个高于”目标，就一定能够实现在全省的位次前移。

未来五年，将是抚顺经济结构明显优化、各种经济竞相发展的五年。我们将优化城乡结构，积极推进城市化进程。我们将优化所有制结构，实现国进民进、两翼齐飞。我们将优化产业结构，促进三次产业协调发展。我们将优化工业内部结构，着力提高工业完整度和丰厚度，重塑工业大市形象。只要我们坚持把发展的着力点放在转变经济发展方式上，做强经济，做优产业，就一定能够增强地区经济发展的活力和动力，就一定能够加快抚顺老工业基地的振兴。

未来五年，将是抚顺开发建设力度空前、城市面貌与生态环境发生巨变的五年。我们将按照现代城市理念建设新城区，结合“退二进三”改造老城区，让城市人居更舒适。我们将修路架桥，纵横东西、贯通南北，让城市交通更便捷。我们将保护好、利用好穿城而过的浑河，打造滨河风景线，让城市景观更靓丽。只要我们坚持不懈地治理大气、污水和垃圾，坚持不懈地还绿、还美于城市，就一定能够将抚顺建设成为点上成景、线上成林、面上成荫、环上成带的绿色生态之城，就一定能够增强城市的吸引力和竞争力。

未来五年，将是百姓收入大幅增加、生活水平明显提高的五年。我们将创造更多的就业机会，让广大劳动者用双手创造财富、改善生活。我们将强化社保体系建设，让更多的群众老有所养、病有所医。我们将增加多种类型的商品房供给，让居者有其屋。我们将大力发展教育、文化、卫生等各项社会事业，让百姓享受更多的改革发展成果。只要我们坚持以人为本，全力保障和改善民生，就一定能够让抚顺人民收入更高、生活更好，就一定能够让千家万户有更多的笑容、更多的温馨、更多的尊严。

经过五年奋斗，到2015年，我市“两城两带”发展格局基本形成，县域和城区经济翻番发展，先进能源装备和化工新材料两大千亿产值基地基本建成，老工业基地全面振兴和资源型城市转型的目标基本实现；新城区建设和旧城区改造基本完成，打造一座宜居、宜业、宜商、宜学、宜游的绿色生态城市的目标基本实现；将改革发展成果更多地惠及百姓，更加注重人文关怀和群众精神生活需求，城乡居民生活水平高于全省平均水平的目标基本实现。届时，我们的抚顺，将成为一个迅速崛起的新抚顺，一个环境怡人的新抚顺，一个抚顺人为之骄傲的新抚顺！

各位代表：今年是“十二五”的开局之年，我们一定要旗开得胜、首战告捷；一定要实现首季开门红、半年双过半、全年“两个高于”的目标。

全市经济社会发展的主要预期目标是：地区生产总值增长16%，地方财政一般预算收入增长27.4%，全社会固定资产投资增长30%，社会消费品零售总额增长16%，城市居民人均可支配收入增长14%，农民人均纯收入增长14%。

完成今年目标任务，重点抓好以下七个方面工作。

（一）深入实施“两城两带”发展战略

继续举全市之力加速建设沈抚新城。核心区，要加快推进万科地产和香港君悦国际城等重点项目，完成以金风湾、玉露潭为核心的景观建设，完成150米生命之环景观地标、20栋城市综合体及总部大厦的主体工程建设。产业区，要加快建设沈东园区和拉古园区，大力发展煤矿安全装备、工程机械装备、石化及输变电装备、新型汽车配套装备四大产业集群，亿元以上项目开工100个、投产50个。生态区，重点推进丰远·热高乐园、泛海拉斐庄园和温泉旅游开发等项目。确保“十一”之前核心区出形象、产业区成规模、生态区上档次。全年沈抚新城生产总值增长50%，财政一般预算收入增长50%，全社会固定资产投资完成300亿元。

高水平规划建设石化新城。高标准编制城市规划和产业规划，打造新的经济增长区和宜居区。核心区和生活区，要加快东洲大街、绥化路北和章党旧城区改造，积极推进名嘉广场和东方清华园等重点项目，大力推进小甲邦和石油机械厂等地块开发，建成龙凤橡胶坝和东洲河橡胶坝。产业区，要重点建设塑料制品、橡胶制品、碳纤维及其制品、精细化工四大产业集群，加快推进方泰7000吨复合材料、碳5和碳9深加工等项目。全年石化新城生产总值增长26.5%，财政一般预算收入增长40%，全社会固定资产投资增长181.7%、达到200亿元。

着力建设南环产业带。结合城区“退二进三”，规划建设企业搬迁的工业园区，筹建南环铁路，重点推进南环公路两侧抚矿集团百万吨再生造纸和40万吨页岩油深加工等项目，加快打造循环经济产业集群。

加快建设浑河景观带。按照生态理念，高标准规划，全方位实施浑河水面和两岸景观、道路、重要节点的改造建设，真正将浑河打造成为一条美丽的河、有灵性的河，真正将浑河两岸建设成为展示抚顺形象的新名片，成为百姓休闲娱乐的好去处。

（二）做大做强工业

坚持工业兴市不动摇，做大存量与引进增量并重，提升传统优势产业与培育战略性新兴产业并举，进一步增强工业对经济的支撑力。

改造提升传统产业。把做强传统支柱产业作为提高产业丰厚度的首要任务，大力发展煤、油、电、钢、铝等传统优势产业，进一步优化工业内部结构和产品结构。煤炭产业，重点推进利用油母页岩资源的煤炭工业转型。石化产业，重点推进石油一厂和化塑厂的搬迁改造，抓好原油集中加工和炼油结构调整，百万吨乙烯项目竣工投产。电力产业，重点推进抚顺发电厂搬迁改造和抚矿集团新建煤矸石热电机组项目。钢铁产业，重点推进抚顺特钢集团百亿技改工程、新钢铁公司环保搬迁、罕王集团大型铸锻件等项目。铝产业，重点推进抚顺铝厂19万吨电解铝二期改造工程。

培育战略性新兴产业。把发展战略性新兴产业作为提高产业丰厚度的重要依托，立足辽宁石油化工大学、抚顺石油化工研究院、煤科总院沈阳研究院，推进产学研合作，加快以企业为主体的科技创新体系建设，培育一批技术先进、市场竞争力强的品牌产品和大型企业。依托沈抚新城，重点发展大型履带起重机、高空作业车和大功率防爆电机等高端装备制造业，积极推进抚顺·山推工业园、抚挖重工工业园建设，打造国家先进能源装备产业化基地。依托石化新城，围绕“千万吨炼油、百万吨乙烯”延长产业链，重点发展碳纤维、改性塑料制品等新材料，打造国家精细化工产业化基地。依托南环产业带，重点发展循环经济，打造世界一流、产业聚集、生态环保的循环经济产业基地。

(三）大力发展县域经济和城区经济

县域经济要全面实施新一轮三年倍增计划，抓紧恢复灾后农业生产和水毁工程，促进新农村建设和农民增收。一是推进现代农业建设。培育壮大农业和畜牧业优势主导产业，扶持建设产业小区,新建食用菌生产小区100个、日光温室小区100个、大型奶牛养殖基地3个,新增设施农业基地666公顷。大力发展农业产业化，对投资规模大、带动力强的龙头企业给予重点扶持。积极推进新网建设，进一步服务好“三农”。加强农业基础设施建设，抓好水库、河道等防洪工程的除险加固，搞好农村水、电、路、生态和人畜饮水工程建设。二是加快发展工业产业集群。清原县输变电配件产业集群企业达到45家，实现销售收入80亿元；新宾县林木加工产业集群企业达到350家，实现销售收入60亿元；抚顺县结合区划调整，规划建设农产品加工和环保产业集群。三是提升县城和重点小城镇规划建设水平。积极推进清原、新宾县城建设，高标准规划和启动抚顺县县城建设。大力推进永陵、南杂木、红透山等重点小城镇建设，积极稳妥地推进农村人口有序向城镇转移。

城区经济要重点围绕三年倍增计划，依托优势，因地制宜，大力发展服务业。新抚区要围绕中央大街改造，加快站前地下广场开发，加快天朗国际广场、东方新天地、万达广场建设，支持浙商国际商贸城、中兴时代广场做大做强，支持高档家居连锁、影视院线及电子信息等新型产业尽快形成规模，打造中央商务区。顺城区要围绕开发6方块、9方块和市政府大楼两侧，建设大型购物中心、五星级宾馆和300米高的标志性建筑，打造城东高端商务区；依托新玛特、乐都汇和北站商业街，打造新华商业圈；积极推进葛布大型城市综合体建设，打造新的商业区。望花区要支持奥特莱广场和商贸大厦等重点商贸企业发展壮大，加快建设特色餐饮一条街、装饰材料一条街，规划建设汽车销售集聚区，启动辽宁石油化工大学计算机广场改造，构筑商业发展新格局。

（四）加快发展民营经济和服务业

加快发展民营经济。全面实施民营经济腾飞计划和中小企业“成长工程”，扶持罕王、抚挖重工、佳化化工3家企业上市。进一步优化发展环境，鼓励全民创业，培育更多抚顺自己的“小老板”。扩大融资渠道，引进私募股权投资机构5家，新增担保公司5家、小额贷款公司5家。全年民营经济增加值增长23%。

加快发展生活性服务业和生产性服务业。生活性服务业要围绕便民利民和扩大消费，加强市场体系建设，重点规划建设蔬菜批发市场和汽车4S店。生产性服务业要围绕大中型工业企业的服务和配套，加快建立煤研和油研的研发基地，重点发展现代物流业和信息产业。

加快发展房地产业。积极推进沈抚新城、石化新城、城东三期、月牙岛两岸、榆林等重点地区房地产开发，加快恒大、万科等地产项目建设。全年开发投资增长22%，商品房销售206万平方米。大力发展建筑业，建筑业增加值增长20%。

加快发展文化旅游业。全力推进沈抚新城文化产业园区建设，培育扶持辽东特艺、艺博雕刻等10户文化产业龙头企业。做大红河峡谷漂流、皇家海洋主题乐园、永陵、三块石等精品旅游项目，积极推进金山石佛、和睦国家森林公园等重点旅游项目建设。组织好春季踏青赏花、夏季激情漂流、秋季登高赏叶、冬季冰雪农家、清前满族文化等特色主题旅游活动，早日将抚顺打造成为全国知名的旅游大市和旅游强市。

（五）狠抓城市规划建设和管理

提高城市规划水平。坚持以规划引领城市建设，加快编制石化新城、旧城区改造等专项规划，重点完善东洲地区、产业园区、采煤沉陷区和高湾地区的控详规划。

加强基础设施建设。续建抚通和桓永高速公路，拓宽高望线和沈通线；完成东环大道、南环路西延和中央大街北延，打通青年路至鞍山路，改造永济路、东林路、煤都路，整治绿化永宁南街。新建海新大桥、东洲河口桥、甲邦桥和月牙岛北岸人行桥，改造章党大桥和东洲桥。全面实施浑河及两岸的河堤景观、带状公园、滩涂公园和防洪堤坝等重点工程，启动东湖岛公园、东洲公园和东林公园建设，建成沈抚新城南北湖公园、月牙岛生态公园和人民广场。全面实施城东三期基础设施大配套工程，改造城市输供水管网15公里、配水管网30公里、供热管网73公里，实现并网供热200万平方米。

加强城市管理。集中整治城市东西出入口、城乡结合部、城区主要街路和中心商业区，治理占道经营和私搭乱建。改善居民居住环境，加大物业管理力度，推进棚改新区环境建设，绿化改造居民小区22个和新开发小区27个。更新公交车50台、出租车500台，延伸公交线路里程50公里。强化城市经营，坚持“先改善环境、后成片开发”的原则，努力提高土地集约利用水平。

加强生态城市建设。围绕创建国家森林城市，大力推进城区造林绿化。全面实施污水处理厂提标改造和新建扩建工程，加强工业污染防治和大伙房水库饮用水源地的保护。综合整治浑河市区段主要支流河，实现雨水、污水分离，污水处理率达到70%。关停演武垃圾场，新建西舍场无害化卫生填埋场，日处理垃圾能力达到1400吨。强化污染物减排，拆除10吨以下燃煤锅炉83台。通过绿化美化、治水治污，让抚顺的天更蓝、水更绿、空气更清新，让抚顺的环境更美好。

（六）进一步深化改革扩大开放

深化国企改革。稳妥推进抚顺特钢集团和抚顺铝厂的政策性破产，完成以解决“一金两险”为重点的142户正在改制和13户尚未改制企业的改制工作。认真解决138户已改制企业职工安置等遗留问题，进一步落实困难企业省市各三分之一补助、按灵活就业补缴保费、退休医保补助等政策，努力维护职工合法权益。稳步推进事转企改制，适时启动大集体改革。支持改制企业“靠大船”和联合重组，使其迅速发展壮大。

扩大对外开放。围绕产业发展规划和资源深加工，重点面向资本密集区、行业龙头企业、中央大企业和世界知名企业，坚持招商与选商、引资与引智、域内与域外并重，大力开展主题概念招商和产业集群招商，努力在市场建设、商贸流通和房地产开发的招商引资上取得新突破。全年实际利用外资增长15%，引进域外资金增长30%，外贸出口增长15%。

积极向上争取。完成国家将抚顺列为全国首家矿山地质灾害综合治理试点市的工作；在争取资源枯竭型城市转型试点市支持政策延续、采煤沉陷区内农民和企业脱险安置政策等方面，取得实质性进展。

（七）更加注重民生和社会事业发展

今年的民生工作，我们要从老百姓最关心、最亟需解决的问题着手，努力实现新突破。继续抓好就业，支持以创业带动就业，城镇实名制就业达到9.5万人，充分就业社区达到80%以上，确保零就业家庭动态为零。继续提高企业离退休人员基本养老金标准，继续提高城镇企业职工医疗保险待遇。继续扩大新型农村养老保险试点范围，清原、新宾实现养老保险全覆盖，惠及农民40万人。积极推进被征地农民社会保障工作。继续推进棚户区改造、农村特困户危房改造、减灾安居等重点工程，改造城市和工矿棚户区20万平方米、旧城区30万平方米，惠及百姓2.5万人。加快推进殡仪馆搬迁改造。继续开展社会救助活动，资助贫困大学生800名，医疗救助贫困家庭3.7万户，对18周岁以下患白血病、先天性心脏病的贫困儿童实施大病救助。继续坚持开发式扶贫，减少农村低收入人口3.8万人，帮扶农民脱贫致富1300户，建设农村单亲贫困母亲安居住房100套，免费为城乡2万名贫困妇女提供健康检查和健康教育。继续建好“菜园子”、抓好“菜篮子”，努力保障市场供给，控制居民消费价格涨幅。继续推进平安抚顺建设，购置142台社区巡逻车，实现夜间巡防全市社区全覆盖，进一步增强群众的安全感，争创“全国综治工作先进市”。继续强化信访稳定工作，全力维护人民群众的合法权益和社会稳定。继续强化生产安全、消防安全和道路交通安全隐患排查治理，严格食品药品安全监管，坚决遏制重特大事故发生。

我们就是要通过更多付出、更多努力，为老百姓解难事、办实事、谋福祉，让老百姓得到实惠、感到温暖、看到希望，让老百姓生活得更舒心、更愉快、更美好。

我们要大力发展各项社会事业。一是深入实施人才培养工程，重点在人才引得进、用得上、留得住等方面有新突破。二是全面贯彻《辽宁省中长期教育改革和发展规划纲要》，推进义务教育均衡发展，重点发展学前教育，大力发展职业教育，新抚区、顺城区率先进入省义务教育均衡发展示范县（区）行列，建设职业教育集团2－3个。三是大力弘扬雷锋精神，加强精神文明建设，争创“全国双拥模范城”；组织开展“红诗红歌”群众文艺调演、月牙岛中秋之夜专场演出、走进沈抚新城歌舞表演等10项大型群众性文化活动；建成抚顺剧院，筹建博物馆、美术馆。四是稳妥推进医药卫生体制改革，扩大城镇居民医疗保险覆盖面，进一步完善基本医疗和公共卫生体系，完成第四医院和第五医院的改造建设。五是广泛开展全民健身活动，举办第三届全民健身节和第六届全民运动会，规划建设东林路体育产业园区，发展壮大体育产业。

继续做好国家安全、烟草、金融、保险、邮政、通信、电力、气象、统计、民族、宗教、司法、外事、侨务、人防、档案、防震、人口和计划生育、老龄、妇女儿童、残疾人等各项工作。

三、建设人民满意政府

人民政府为人民。人民群众的所思、所想、所盼，就是我们工作的目标和努力的方向。我们一定要在思想上尊重群众、感情上贴近群众、工作上服务群众、发展上为了群众，不断加强自身建设，更好地肩负起振兴抚顺的使命，更好地担当起富民强市的重任。这是我们的职责所在，我们必须做到，我们也一定能够做到。

要更加注重发展。加快发展是我们解决各种矛盾的根本途径。我们将始终坚持发展第一要务不动摇，心无旁骛，遵循发展规律、创新发展理念、凝聚发展合力、破解发展难题，聚精会神搞建设，一心一意谋发展。我们将千方百计抓住机遇用好政策，千方百计搞好服务大上项目，千方百计引进资金引进人才，千方百计鼓励创新支持创业，早日实现抚顺的大发展、快发展。

要更加注重落实。巩固“三讲一鼓劲”活动成果，满怀激情，亲历亲为，真抓实干，不断提高创造力、决策力和执行力。要建立健全激励机制和约束机制，真正让那些埋头苦干、扎实工作、成绩突出的人，在政治上有待遇、物质上得实惠、荣誉上受表扬。要走进矛盾，面对困难，切实解决重点、难点和热点问题。要简化工作流程，提高工作效率，坚持事不过夜，定了就办、办就快办、办就办好。

要更加注重依法行政。认真贯彻国务院《全面推进依法行政实施纲要》，加强和改进制度建设，强化行政监督和问责，努力提高科学决策、民主决策、依法决策的水平。主动接受市人大及其常委会的法律监督、工作监督和市政协的民主监督，认真办理人大代表建议和政协提案，虚心听取各民主党派、工商联、人民团体和社会各界的意见和建议，自觉接受群众监督，广泛凝聚社会各方面的力量和智慧，使政府各项工作经得起实践、人民和历史的检验。

要更加注重清正廉洁。强化教育、制度、监督并重的惩治和预防腐败体系建设，牢固树立贪污受贿是腐败，不干事、不作为也是腐败的理念，努力营造激情干事光荣、平庸无为可耻的氛围。加强对国有资产管理、政府采购、土地出让、工程建设招投标、项目规划审批等重点领域的审计和监察，严厉打击权钱交易、以权谋私、商业贿赂等腐败行为，打造廉洁政府。

各位代表：加快发展的历史重任需要我们奋发有为，你追我赶的竞争态势激励我们拼搏进取，振兴抚顺的神圣使命更使我们别无选择。让我们在市委的坚强领导下，深入贯彻科学发展观，以昂扬的斗志、旺盛的激情、必胜的信心、扎实的工作，为加快抚顺的发展振兴，为抚顺人民生活得更加美好而努力奋斗!
